# Editorial Responsibility

**Published:** 1885-12

**Authors:** Geo. H. Cushing

**Affiliations:** Chicago, Illinois


### EDITORIAL RESPONSIBILITY.

BY DR. GEO. H. CUSHING, CHICAGO, ILLINOIS.

The editorial in the October number of the INDEPENDENT PRACTITIONER upon this theme, furnishes me an excuse for saying what I have long felt needed to be said, but which I have hesitated to present, because doubtful if it would accomplish any good.

I think it is generally supposed that the editor of a scientific or professional journal is fully informed regarding the various matters pertaining to the subjects naturally presented therein, both as to well established facts and generally accepted theories, and also as to the various unsettled theories which may be matters of discussion. I believe, also, that a large proportion of the readers of dental journals unthinkingly accept as reliable, almost anything that appears therein on subjects with which they are not familiar. In view of these premises, I think that the editor should be held to a more strict accountability than he now is, and I desire briefly to state my idea of the extent and nature of his "editorial responsibility."

First, as to correcting his correspondent's manuscript. He certainly should not only be allowed, but *expected* to make such corrections as may be necessary to render the article grammatical, the construction of sentences correct, or to make more plain the obvious meaning of the author. There are very few writers for the journals who do not commit more or less errors of the kind referred to, in almost every communication they offer, and they should be only too thankful to the editor who would save them from the adverse criticism the publication of such errors would be sure to entail. This is almost entirely a matter of taste, and is not important as bearing upon the value of a journal otherwise properly edited, but one province of the editor certainly should be to place all communications before his readers in the best possible shape, so that he does not change the sense intended to be conveyed.

Now, as to the responsibility which I regard as vital in making professional journals of the greatest value. It should be the duty of the editor, when any correspondent states what is known to be erroneous or misleading, to correct such statements in a note, inclosed in brackets; or when a position is assumed as to certain views being generally accepted, if the editor knows they are not, he should also so state in the same way, and where any opinion is advanced the editor might briefly suggest the opposite side of the argument. These notes or comments should occur in the body of the article. In addition to this, the editor should write an article briefly reviewing all the communications in the journal, and in case of anyone of particular importance, he should devote a special article to that. In this way our dental journals could be made much more valuable to the general reader than they now are; thought would be stimulated, and a more general and intelligent discussion be induced. This method would not constitute the editor an "arbitrary censor," in any offensive sense, nor make the journal "to reflect only his own opinions."

I do not mean by the above to insist that a man has no right to edit a journal in the way in which journals are now generally edited, but that the course I have briefly described is the only one which can secure the most valuable service to the profession.

